# Multiple primary tumours: incidence estimation in the presence of competing risks

**DOI:** 10.1186/1478-7954-7-5

**Published:** 2009-04-01

**Authors:** Stefano Rosso, Lea Terracini, Fulvio Ricceri, Roberto Zanetti

**Affiliations:** 1 Piedmont Cancer Registry – CPO, Turin, Italy; 2 Department of Mathematics, University of Turin, Italy; 3 Unit of Epidemiology and Modelling, Institute for Scientific Interchange (ISI) Foundation, Turin, Italy; 4 Department of Genetics, Biology and Biochemistry, University of Turin, Italy

## Abstract

**Background:**

Estimating the risk of developing subsequent primary tumours in a population is difficult since the occurrence probability is conditioned to the survival probability.

**Methods:**

We proposed to apply Markov models studying the transition intensities from first to second tumour with the Aalen-Johansen (AJ) estimators, as usually done in competing risk models. In a simulation study we applied the proposed method in different settings with constant or varying underlying intensities and applying age standardisation. In addition, we illustrated the method with data on breast cancer from the Piedmont Cancer Registry.

**Results:**

The simulation study showed that the person-years approach led to a sensibly wider bias than the AJ estimators. The largest bias was observed assuming constantly increasing incidence rates. However, this situation is rather uncommon dealing with subsequent tumours incidence. In 9233 cases with breast cancer occurred in women resident in Turin, Italy, between 1985 and 1998 we observed a significant increased risk of 1.91 for subsequent cancer of corpus uteri, estimated with the age-standardised Aalen-Johansen incidence ratio (AJ-IR^stand^), and a significant increased risk of 1.29 for cancer possibly related to the radiotherapy of breast cancer. The peak of occurrence of those cancers was observed after 8 years of follow-up.

**Conclusion:**

The increased risk of a cancer of the corpus uteri, also observed in other studies, is usually interpreted as the common shared risk factors such as low parity, early menarche and late onset of menopause. We also grouped together those cancers possibly associated to a previous local radiotherapy: the cumulative risk at 14 years is still not significant, however the AJ estimators showed a significant risk peak between the eighth and the ninth year. Finally, the proposed approach has been shown to be reliable and informative under several aspects. It allowed for a correct estimation of the risk, and for investigating the time trend of the subsequent cancer occurrence.

## Introduction

During the last decades, improvements in medical and surgical treatments have substantially increased the chances of surviving from a cancer. Cancer survivors now amount to more than 3.5% of population in the US [1], and about 3% in Western Europe [2]. Now more cancer survivors face the problem of subsequent cancers possibly related to the late effects of treatments or to a common etiology of first and subsequent cancers. As for other epidemics in the past, the challenge is towards a research effort to address, and possibly prevent, those elements that increase the chance of developing a second tumour. And, as in the past, the starting point is to correctly estimate the incidence of multiple primary tumours on a population basis.

First of all, there is a problem of differential diagnosis, when it comes to distinguish between local and distant metastases, recurrences and the onset of a truly new lesion. Classifications may also vary leading to substantial differences in rates. For example SEER rules [3] differ substantially from those adopted by IARC [4]. Timing of multiple primaries is also important, as they can occur at the same time (synchronous) or after a time lag (metachronous). Usually synchronous tumours are excluded from analyses, in the belief that they rather represent prevalent silent tumours come to evidence during diagnostic procedures.

Secondly, it should be taken into account that incidence rate of multiple primaries is conditional to the probability of surviving the first tumour, having accumulated sufficient time for developing another one. Usually, the studied statistics is the ratio between observed and expected multiple metachronous primary tumours. Expectation is taken calculating the person-years observed in the cohort of patients with first tumour, applying general population incidence rates. In this way, a group of 100 patients with a short survival of 1 year is equivalent to 10 patients surviving for 10 years. But we know that rarely these two groups with such a different survival experience can be compared for several aspects, even without difference in age distribution. On the contrary, when conditioning on survival probabilities, we get the same number of expected cases only when the overall survival is equal to that of the general population. This assumption holds true only for those pairs where the first tumour has a rather benign course with no substantial influence on the whole survival.

Some of these aspects are not new in the literature, and they were discussed for deriving expected number of deaths (or events) for SMR. Keiding offered a historical perspective of this [5], also showing how one of the oldest statistical techniques was connected to conditional survival probabilities and parametric models. The estimation of the expected number of subsequent cancers adds some complications to the traditional model and should be approached in the framework of competing risks, as many subjects are withdrawn from the population at risk, as time goes by, by death or censorship. Previous works had already shown how the traditional Kaplan-Meier estimator is inappropriate in the presence of competing risks [6], but until now a correct approach taking into consideration competing risks has not yet been applied to the estimation of multiple tumours expected number. In addition, it is also important to consider the time elapsing dimension, as subsequent tumours are often more frequent in the first years, and then decrease, with a later rise after five to eight years depending on the tumour type [7,8].

These aspects of the problem of estimating probabilities of subsequent tumours occurrence in the presence of time-varying rates led us to consider a non-parametric approach based on multi-state models, which can appropriately describe situations where there are several competing outcomes in a time process. Various types of multi-state models have been proposed for analysing multiple end-points in different situations; from transplants to clinical trials and from pregnancy-birth model to infectious disease epidemics (for a review see: [9]). Indeed, we made use of a stochastic process for estimating the risk of developing a subsequent cancer, following the enlightening suggestions offered by Aalen and Gjessing in their work [10].

## Methods

### Statistical methods

**Figure 1:**
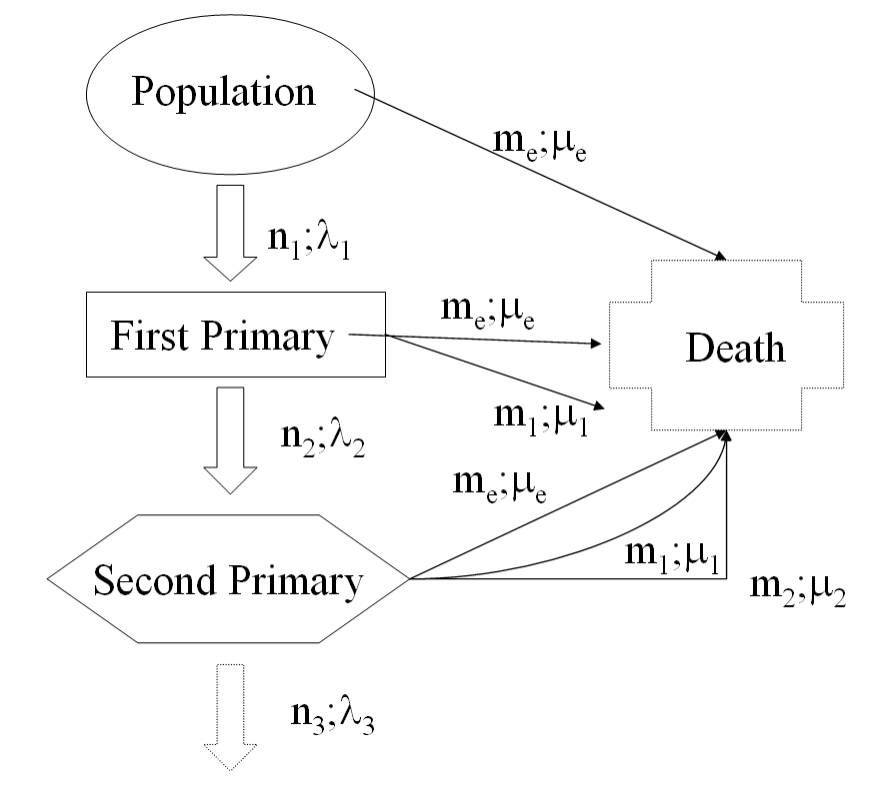
**The multi-state model**.

The tumours occurrence in a general population can be depicted as in Figure 1. From demographic sources we know the amount of deaths (*m*_*e*_) and the general mortality rate (*μ*_*e*_). From cancer registries we measure the number of first primary tumours (*n*_1_) the incidence rate (*λ*_1_), the number and rates of these patients deceased for other causes (*m*_1*e*_;*μ*_*e*_) and for the specific cause of death (*m*_1_;*μ*_1_). After this first process has taken place we can observe (*n*_2_) second primaries with a rate (*λ*_2_) to be estimated conditioned to the quantities and parameters previously seen.

**Figure 2:**
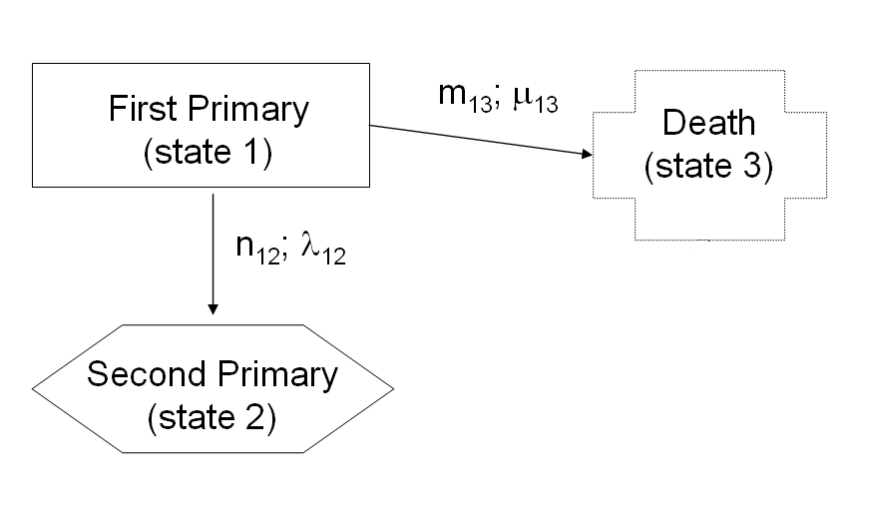
**The simplified Markov model**.

Since we were interested in estimating rate of occurrence after first primary only, we dealt with the simplified model given in Figure 2. This last one satisfied Markov assumption, since it did not take into consideration the past transitions from health state to first tumour. Indeed, as long as we adopt a simplified model as the one in figure 2, Markov condition is satisfied. Of course, the risk of a subsequent tumour can depend not only on subject's age, and his/her current state, but also, for example, on the therapies adopted for treating the first primary tumour. In a more sophisticated version of such model these factors may be introduced as covariates in the transition probabilities.

We applied Markov theory to the process occurring to the first tumour cohort with two different irreversible and reciprocally exclusive outcomes: death and second tumour occurrence. Of course, to calculate transition intensities in this model it was necessary to also consider censored observations. We estimated transition intensities by Nelson-Aalen estimators; then we calculated occurrence probabilities conditioned to different events (occurrence of a second cancer,  (0, *t*) death,  (0, *t*)) in each time interval with the Aalen-Johansen [11] method (**AJ**) in the framework of a Markov process (for details see Appendix). The proposed model is a simple version of the competing risk model on which a vast literature already exists (see, for example, Satten and Datta for marginal estimation of multi-state models with right-censored data [12]).

In a population of size *N*, we calculated the cumulative number of transitions (to second tumour) at the end of the fourteen years period, as:



We then compared it with the number of expected transitions under the assumption of a Markov model with constant transition intensities equal to those expected from the general population.



where  (0, 14) is the cancer occurrence probability in the general population.

So we can derive an incidence ratio for this model:



It must be noted that imposing the same censorship mechanism in calculating expected cases resulted in a less biased estimator, since the same bias originated by the censorship mechanism was at work both in the numerator and in the AJ-IRdenominator.

We calculated 95 percent confidence limits using the AJ variance in formula 7 presented in the Appendix.

### Age standardisation

Since rates of first and second tumours strongly depend on age, analysis must be done in age strata or a standardisation procedure must be defined. We pursued both strategies grouping age at breast cancer occurrence in five classes: 0–44, 45–54, 55–64, 65–74, 75+. A standardised AJ estimator for the whole population can be obtained as follows:

• For each age class *k *we calculated the  AJ estimator : let *N*_*k *_be the number of subjects in class *k *at time 0 and set a weight , where *N *equals the sum of the *N*_*k*_'s;

• define ;

• under the assumption that weights are deterministic variables,



• so: 

### Simulation study

We first carried out a simulation study for validating the proposed model. Our aim was to compare the estimated number of second tumours using the AJ estimator with that obtained with the person-year approach in a simulation. This comparison can be better performed simulating the process of second tumour occurrence taking under control biasing factors such as censoring. We then simulated different dynamics of second tumour occurrence. We considered a simulated cohort of 10000 patients with a first primary and with same age and period of incidence, followed up for 10 years. We imposed a survival exponential law with a constant mortality rate of 0.2. Firstly, occurrence of a second primary was kept constant for the whole follow-up period. We compared the simulated number of second tumours to the number estimated both by the person-year, and by the AJ approach. We let the second tumour incidence rate vary from 0.00025 to 0.004, corresponding to a rate ratio of 0.25, 0.5, 1, 2 and 4. Then, the effect of standardisation by age was investigated repeating the simulation for the five age classes (each with the same number of subjects), varying the occurrence rates, but always keeping them constant for the whole period. Secondly, the simulation was extended to situations where also occurrence rates varied in time: at a constant decreasing or increasing trend, or in a bimodal way. Age standardisation was then applied on bimodal rates simulation.

The simulation engine was based on random chains of multinomial probabilities *M *(*P*_*α*_, *P*_*β*_, *P*_*γ*_) at each time-click *t*, with the constraint that *P*_*α *_+ *P*_*β *_+ *P*_*γ *_= 1. *P*_*α *_and *P*_*β *_are normally distributed iper-parameters in a three class model, respectively representing the probability of transition from steady state to second tumour and transition from steady state to death.

### Subjects

After the simulation study, we applied the Aalen-Johansen model to the incidence data from the Piedmont Cancer Registry (RTP). RTP collects all incident tumours in the resident population (about one million inhabitants) of Turin (Italy) since 1985. We selected all first occurrences of breast cancer (following IARC rules, cases occurring in the paired breast gland were excluded). We included all cases diagnosed up to 1998 and then we prolonged the observation period for detecting subsequent tumours up to the end of year 2000, allowing for a reasonable amount of follow-up time also for the last incident cases. We excluded all cases diagnosed with Death Certificate Only (DCO), skin cancers other than melanoma and all synchronous tumours.

## Results

### Simulation study

With a sample size of 10000 subjects replicated for 1000 times we had an average of 55.1 cases with a second tumour in ten years, given a constant annual death rate of 0.2 and a constant annual second tumour rate of 0.001. These were estimated as 53.00 cases by the AJ estimator and as 44.4 cases using the person-year approach (Table 1). Varying the base rate from 0.00025 to 0.004, the percentage of bias in the AJ estimators stayed between 3.81% to 5.80%, while the bias in the number of estimated cases using the classical amount of person-years ranged between 19.06% to 19.73%. Age-standardisation averaged the observed bias with 4.75% for the AJ estimator and 19.42% for the classical approach. In Table 2 we presented the effect of varying the base incidence rate by follow-up time. We observed a wider bias, that, however, when averaged by age-standardisation, was kept reasonably low (4.67%) for the AJ estimator and 4.93% for the person-year approach. The bias was the lowest (+1.25%) in the case of constantly decreasing rates. Indeed, the cumulative effects of competing mortality are larger as time goes by and the bias reached a 14.7% for the AJ estimators, and a 26.3% calculating the estimated cases from the average person-years, in the case of a constantly increasing rate. However, the most frequent case when dealing with subsequent primary malignancies is the situation where the rate is constantly decreasing or bimodal. We must note that the occurrence of a constantly increasing rate is quite uncommon in the real situation.

**Table 1:** Simulation Study, I

Averages over 1000 simulation runs	0.00025	0.0005	0.001	0.002	0.004	Age-stand.
Number of simulated cases	13.8	27.6	55.1	109.34	218.5	82.5
Number of estimated cases(Aalen-Johansen)	13.0	26.0	53.0	104.0	208.0	78.6
*Bias %*	5.80	5.80	3.81	4.88	4.81	4.75
Number of estimated cases(person-year)	11.1	22.3	44.4	88.5	175.4	66.5
*Bias %*	19.25	19.34	19.34	19.06	19.73	19.42

**Table 2:** Simulation Study, II (^1^rates = 0.002; 0.0018; 0.00165; 0.0015; 0.00135; 0.0012; 0.001; 0.0008; 0.00065; 0.0005, ^2^rates = 0.0005; 0.00065; 0.0008; 0.001; 0.0012; 0.00135; 0.0015; 0.00165; 0.0018; 0.002, ^3^rates = 0.001; 0.002; 0.001; 0.0005; 0.0005; 0.0005; 0.001; 0.002; 0.001; 0.0005, ^4^rates for age groups = annual rates by increasing Relative Risks {0.25; 0.5; 1; 2; 4})

Averages over 1000 simulation runs	Constantly decreasing^1^	Constantly increasing^2^	Bimodal^3^	Age-stand.^4^
Number of simulated cases	83.95	52.81	59.41	88.56
Number of estimated cases(Aalen-Johansen)	85.00	45.00	57.00	84.42
*Bias %*	1.25	14.79	4.06	4.67
Number of estimated cases(person-year)	66.46	66.72	53.31	84.19
*Bias %*	20.83	26.34	10.26	4.93

### Risk of a second tumour following breast cancer

We identified 9233 women with breast cancer in Turin from 1985 to 1998; 249 cases were excluded as they were identified only from death certificate (DCO), and 58 cases were excluded since they were synchronous cancers, leaving 8926 cases for analysis. From this cohort, 353 second (metachronous) primary tumours (excluding skin cancers) developed during the prolonged follow-up period (1985–2000). The completeness of clinical documentation was rather high, considering that registries work on a population basis, with a 94.5 percent of microscopic confirmation for first tumours that reached 99.3 percent for second tumours. In Table 3 we presented the observed number of second tumours by site and the AJ-IR^stand^ that assumed both observed and expected probabilities fully conditioned to survival.

**Table 3:** Number of observed second tumours in a cohort of women with breast cancer in Turin (Italy), AJ-IR

Cancer Site	Observed cases	AJ-IR^stand ^(95% C.L.)
Mouth Pharynx	7	0.80 (0.39–1.47)
Oesophagus	5	2.38 (0.92–5.01)
Stomach	29	1.38 (0.97–1.93)
Colon-Rectum	66	0.87 (0.69–1.10)
Liver	7	0.61 (0.23–1.30)
Gallbladder	8	0.78 (0.38–1.40)
Pancreas	20	1.39 (0.89–2.07)
Lung	24	0.80 (0.54–1.15)
Melanoma	14	1.15 (0.65–1.88)
Cervix uteri	9	0.68 (0.34–1.22)
Corpus uteri	54	1.91 (1.47–2.44)
Ovary	24	1.12 (0.74–1.64)
Bladder	14	0.74 (0.42–1.21)
Brain & NS	4	0.56 (0.18–1.31)
Thyroid	11	1.00 (0.46–1.89)
NHL	21	1.32 (0.85–1.94)
Leukaemias	9	0.81 (0.39–1.49)
Other & unspecified	27	0.48 (0.33–0.68)
Total (breast and skin excluded)	353	0.99 (0.91–1.10)

A significant risk increase was observed only for corpus uteri cancer, while the only cancer site with a reduced statistically significant risk was "other and unspecified". In addition, AJ-IR^stand^ showed a suggestive risk increase also for cancers of oesophagus, stomach and pancreas, and for non-Hodgkin lymphoma, although confidence limits still included unity. In particular the increased risk of a subsequent cancer located in the anatomical sites of oesophagus, stomach, lung or thyroid was suggestive of a late effect of local radiotherapy of the breast tumor. Grouping together these cancers, we observed a total of 69 patients, and the estimated AJ-IR^stand ^was 1.15 (95% C.L.: 1.04–1.28).

**Figure 3:**
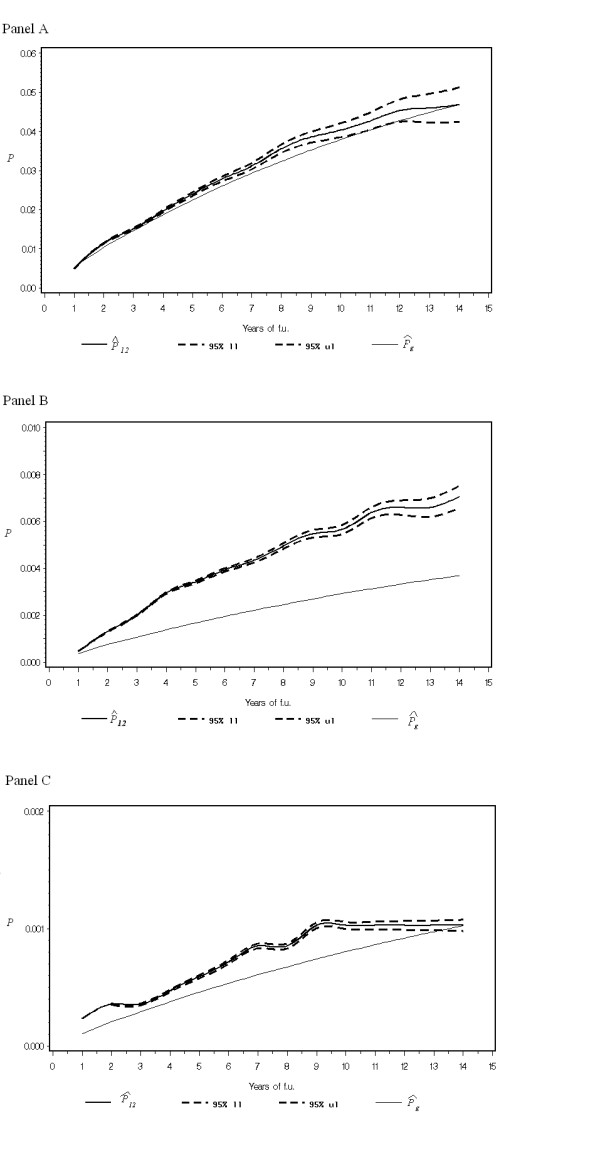
**Cumulative probability of a second tumour after breast cancer.** Panel A: All cancers (excluding skin cancer). Panel B: Corpus Uteri. Panel C: cancers related to radiotherapy (oesophagus, stomach, lung and thyroid gland).  is the cumulative observed probability of a second tumour in the cohort of patients with a primary tumour, with its 95% upper (95% ul) and lower (95% ll) confidence limits.  is the cumulative estimated probability of a second tumour assuming a constant intensity taken from the general population.

Another interesting feature of the AJ estimator is the possibility of studying the dynamic of second primary occurrence over time. In Figures 3, we presented the time trend of the  AJ estimator,  (0, *t*), which is the estimated cumulative probability of a second primary until time *t *for all cancers (Panel A), for cancer of corpus (Panel B), and for cancers related to breast cancer radiotherapy (Panel C). In particular, Figure 3 showed that there was a relative increase of probability of developing a second primary from the fourth to the tenth year after diagnosis of a breast cancer, even if the cumulative AJ-IR for all cancers was not significant. For corpus uteri the Figure 3 (Panel B) showed a persistent increase of risk across all the observation period. Figure 3 (Panel C) showed an increased risk after 5 years of follow-up for radiotherapy-related cancers with a peak at 8 years.

**Figure 4:**
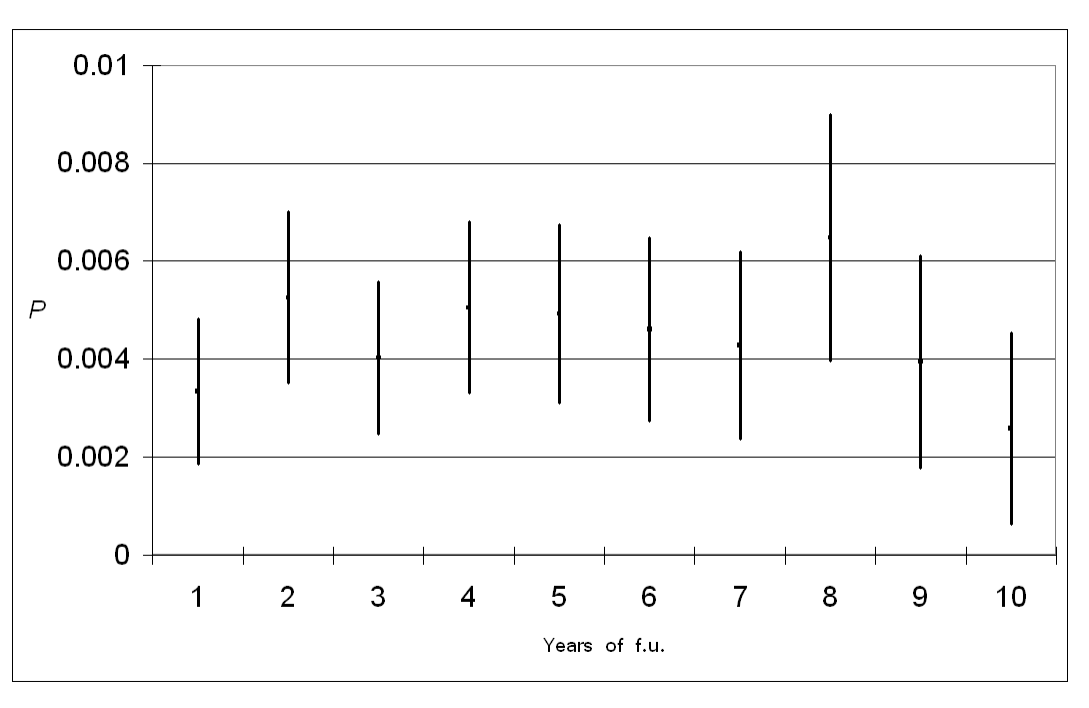
**Interval probabilities of a second tumour after breast cancer during the first ten years of follow-up and 95% confidence bars**.

Within this method, it is also possible to provide additional insights on the variations in occurrence probabilities over time. The mean interval time between two tumours (calculated over the observed period of 14 years) was 5.43 for all type of tumours, a little shorter for corpus uteri (5.27), and notably longer (6.07) for cancers related to radiotherapy (Table 4). For all cancers and for corpus uteri the time interval between the two tumours increased over age, being the shortest (about 2 years) for patients younger that 45 years of age. In figure 4 we also investigated when probabilities peaks occurred: we found two peaks at 2 and 8 years. The second peak at 8 years was mainly sustained by cancers related to radiotherapy and a AJ-IR^stand ^value of 1.29 (95% C.L.: 1.25–1.33) was observed at the eighth year of follow-up.

**Table 4:** Mean time (years) occurrence of a subsequent primary cancer in a cohort of women with breast cancer

**Subsequent Cancer Site**	**0–44**	**Age** **45–54**	**Groups** **55–64**	**65–74**	**75+**	**Age-Standardised**
**All Sites**	5.10	5.14	5.36	5.49	6.17	5.43
**Corpus uteri**	2.03	4.99	4.85	5.52	7.69	5.27
**Cancers related to radiotherapy**	--	7.33	5.21	5.81	6.96	6.07

## Discussion

The occurrence of subsequent primary tumours can be due to several factors. Subsequent malignancies can initially result from intense clinical surveillance after the first tumour; they can occur later on as therapies for the first primary can induce carcinogenesis. Finally, they can also be due to shared risk factors, including environment, life styles and inherited genes predisposing to higher susceptibility. However, the high fatality of several cancers hinders the possibility of observing subsequent events, even if their probability is sensibly increased. Following the suggestions of Hougaard [9], we applied a simple Markov model for competing risks and we studied the transition probabilities from first to second tumour varying in time. The observed time trend of second primary occurrence is often not constant with two or more waves of increased risks during the observed period. For this reason we resorted to a non-parametric approach, directly calculating AJ estimators. Some other possible parametric or non-parametric approaches could be based on a piecewise constant hazard function [13], or on stratification by age or other covariates when proportional hazard model cannot be used [14], or multivariate space-state models [15]. However, lack of available clinical information and shortness of time series in population-based series of disease occurrence, as in the case of cancer registry data, hinders the possibility of fully exploiting the power of more complex models.

Simulation showed how AJ estimators led to less biased estimates than the person-year method. This is essentially due to the fact that the AJ estimators are built up taking into consideration in numerator and in denominator the exact amount of transitions and person-time at risk at each time interval. On the contrary, the person-year method calculates denominators only at the end of the observation period. Moreover, not only can AJ estimators give a more precise result at the end of the period, but they also describe the full probability trend over time. When keeping the incidence rate constant over the period, the person-year approach led to a larger bias, underestimating the number of events. Also the AJ estimator had some limitations; however, the bias was within the 5% error probability as shown in tables 1 and 2 by the simulation.

The situation is even more complicated with varying rates. The largest bias was seen with constantly increasing rates. In this case, the person-year approach gave rise to a larger than simulated number of events, while the AJ estimator underestimated the overall number of events, although to a lesser extent. This limit is due to the unavoidable introduction of discrete time intervals in the analysis of an intrinsically continuous dimension. In the real situation a constantly increasing occurrence rate is quite uncommon, and usually an early increase risk followed by a decrease or a late peak of incidence is observed. We therefore concluded that we could apply the method of AJ estimators for analysing subsequent occurrence of cancers after a primary breast cancer.

Applying the method to the Turin data, we observed an increased risk of a cancer of the corpus uteri, as also observed in other studies [16-20], although with lower values, and it is usually interpreted as the common shared risk factors such as low parity, early menarche and late onset of menopause. The most remarkable finding of our study was an increased risk of oesophagus, stomach, lung and thyroid cancer.

Indeed, we observed a significant increased risk, grouping together those cancers possibly associated to a previous local radiotherapy: the AJ_12_(s, t) estimators showed a significant risk peak between the eighth and the ninth year. This was suggestive of a late effect of local radiotherapy of the breast tumour. Other studies [18,20], based on larger populations and longer follow-up, observed the association of breast cancer with oesophagus, stomach, lung and thyroid cancers. On the other hand, treatment for early-stage invasive breast cancer shifted in the 1990s from radical mastectomy substantially without regional radiotherapy to increasing use of breast-conserving surgery followed by breast radiation (post-lumpectomy radiation [21]). In conclusion the proposed approach has been shown to be valid and informative under several aspects. It allowed for a reliable estimate of the number of events, conditioning to observed survival. It also allowed description of the changing pattern of risk over time. Future developments of the method should be directed to the parametric modelling of transition probabilities, also in relation to clinical or epidemiological explanatory variables.

## Appendix

### Markov models and Aalen-Johansen estimators

Markov models deal with situations where individuals can belong to a finite set of states and move to one state to some others with a probability, possibly depending on time. The main hypothesis (the Markov assumption) is that the probability of moving from state *i *to state *j *at time *t *depends only on *i, j *and *t *and not on the previous states.

For every possible move *i *→ *j *it is defined a *transition intensity map *from state *i *to state *j*, *α*_*ij *_(*t*), and a *cumulative intensity map * Then we define the *probability transition maps *and the *probability transition matrix *in the interval [*s*, *t*]



The key mathematical ingredient for estimating probability transitions maps is the following formula which is derived from Chapman-Kolmogorov equation for Markov models:

(1)

where *u*_1 _< ... <*u*_*r *_is a partition of [*s*, *t*] and the limit is taken for *k *tending to infinity and the interval lengths tending to zero; **A **= {*a*_*ij*_} is the upper triangular matrix defined by

(2)

In the presence of right censoring, the cumulative intensity maps can be estimated by using the Nelson-Aalen estimators (**NA**), as follows. For every time *t*, let *N*_*ij *_(*t*) be the number of transitions from state *i *to state *j *in the time interval [0, *t*], and *Y*_*i*_(*t*) be the number of individuals which are in state *i *at time *t*. Then the *Nelson-Aalen estimator*, giving an estimation for the cumulative intensity, is

(3)

The estimation of the probability transition matrix can be obtained by using the values of Nelson-Aalen in formula (1). We get in this way the Aalen-Johansen (**AJ**) estimator for the probability transition matrix:

(4)

For a complete reference see the book on statistical models based on counting processes by Andersen et al [11].

### Application to multiple primary tumours

We assumed that the starting time, 0, is the time of diagnosis of the first tumour for each individual.

We construct a simple model with three states

1 first tumour

2 second tumour

3 death after a first (but not a second) tumour

where 2 and 3 are absorbing states and the possible moves are

1 → 2, 1 → 3.

In order to fit our situation in a Markov model, we need to make sure that every individual goes through at most one move in every time unity. This is the mathematical reason for eliminating all sinchronous situations.

According to definitions 3 and 4 we set for every move *i → j*:

(5)

The estimation of the probability transition matrix can be obtained by approximating the integral product in formula 4 via time discretisation. We get in this way the **AJ **estimator for the probability transition matrix:

(6)

where  is the matrix obtained by formula 2 with the estimated values.

The **AJ **estimators are consistent and valid also with right censoring and when the underlying process is non-Markovian [22].

Following [11] we estimated the variance as:

(7)

## Competing interests

The authors declare that they have no competing economic or financial interests.

## Authors' contributions

SR conceived the idea for the study. SR, FR and LT planned and designed the research. LT developed the statistical models. FR analysed the data. SR and RZ wrote the first draft of the manuscript. RZ coordinated the project. All authors edited and approved the final version of the manuscript.
